# P-1969. Safety, Immunogenicity, and Pharmacokinetics of Repeat Dosing with AZD7442 (Tixagevimab/Cilgavimab): Final Analysis of the ENDURE Phase 2, Dose-Ranging Study in Immunocompromised Participants

**DOI:** 10.1093/ofid/ofae631.2128

**Published:** 2025-01-29

**Authors:** Taylor Cohen, Marimer Rensoli Valzquez, Chigo Munthali, Herve Tchouakam Kouekam, Sam Matthews, Audrey Sharbaugh, Rohini Beavon, Dilki Wickramarachchi, Sharon Otal, Haitao Yang, Anastasia A Aksyuk, Lindsay E Clegg, Huixia Zhang, Antonella Nadia Tuillio, Seth Seegobin, Katie Streicher, David Wheeler, Lee-Jah Chang, Hugh Montgomery

**Affiliations:** AstraZeneca, Gaithersburg, Maryland; Charisme Medical and Research Center, Miami Lakes, Florida; AstraZeneca, Gaithersburg, Maryland; AstraZeneca, Gaithersburg, Maryland; AstraZeneca, Gaithersburg, Maryland; AstraZeneca, Gaithersburg, Maryland; AstraZeneca, Gaithersburg, Maryland; AstraZeneca, Gaithersburg, Maryland; AstraZeneca, Gaithersburg, Maryland; AstraZeneca, Gaithersburg, Maryland; Translational Medicine, Vaccines & Immune Therapies, BioPharmaceuticals R&D, AstraZeneca, Gaithersburg, MD, USA, Gaithersburg, MD; AstraZeneca, Gaithersburg, Maryland; AstraZeneca, Gaithersburg, Maryland; AstraZeneca, Gaithersburg, Maryland; AstraZeneca, Gaithersburg, Maryland; AstraZeneca, Gaithersburg, Maryland; CARE-ID, Annandale, Virginia; AstraZeneca, Gaithersburg, Maryland; University College London, London, England, United Kingdom

## Abstract

**Background:**

Prior studies of the SARS-CoV-2 monoclonal antibody (mAb) combination AZD7442 (tixagevimab/cilgavimab) included limited numbers of immunocompromised (IC) participants, the target population for COVID-19 pre-exposure prophylaxis. We report repeat dosing data of AZD7442 in an IC population.

**Figure:**
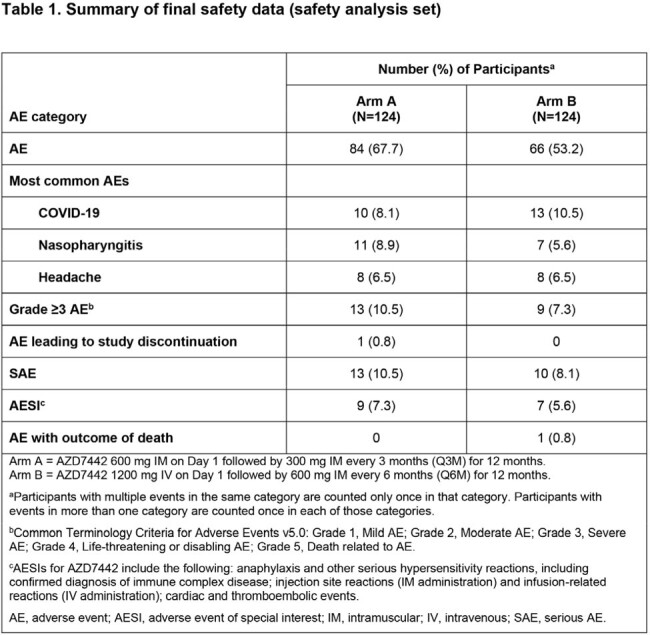

**Methods:**

The randomized, open-label, ENDURE study (NCT05375760) enrolled individuals aged ≥ 18 yrs, weighing ≥ 40 kg, moderately to severely IC or at risk of inadequate vaccine response, and SARS-CoV-2–negative at baseline. Participants were randomized 1:1 to AZD7442 600 mg intramuscularly (IM) on Day 1 + 300 mg IM every 3 months (Arm A) or AZD7442 1200 mg intravenously on Day 1 + 600 mg IM every 6 months (Arm B). Month-12 dose was not administered due to non-safety related trial termination. Primary objectives were safety and immunogenicity (anti-drug antibodies; ADAs); secondary objectives were pharmacokinetics (PK) and SARS-CoV-2–neutralizing antibody (nAb) titers.

**Figure:**
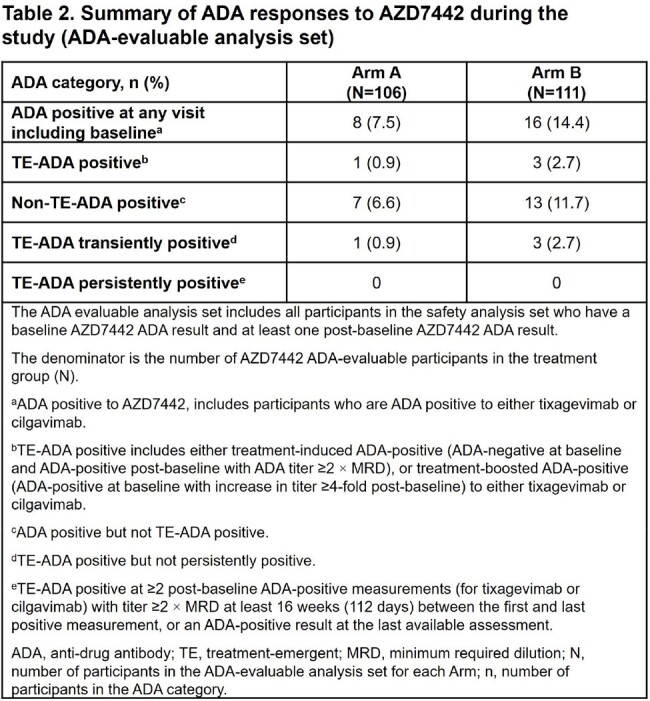

**Results:**

Of 251 randomized participants, 124 were dosed in Arms A and B. Median exposure duration was 373.5 days in both arms. Participants’ mean age was 55.7 yrs, 53.2% were female, and 81.9% had not received a COVID-19 vaccine prior to study start. Adverse events (AEs) occurred in 84 (67.7%) participants in Arm A and 66 (53.2%) in Arm B. The proportion of participants with serious AEs (overall 9.3%) and AEs of special interest (3.1%) was similar across doses and following repeated dosing (Table 1). ADA incidence (% treatment-emergent ADA+) was similar in both arms (Arm A, 0.9%; Arm B, 2.7%) with no apparent impact of ADAs on safety, PK, or SARS-Cov-2 nAb titers (Table 2). Following dosing, mAb serum concentrations were stable in Arms A and B, with no accumulation observed (Figure 1). A strong correlation (R > 0.78) was observed between pseudovirus nAb titers and AZD7442 serum concentrations for both arms (Figure 2).

**Figure:**
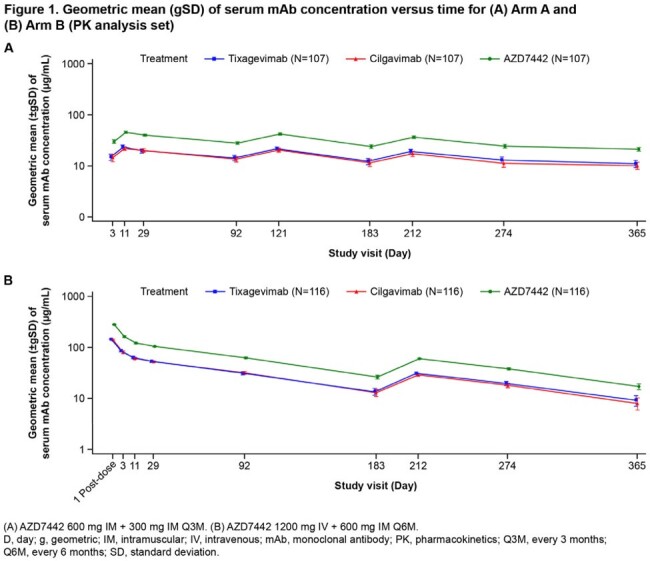

**Conclusion:**

AZD7442 safety and ADA profiles were consistent across doses and dosing regimens. No accumulation in serum AZD7442 concentrations was observed with repeat dosing compared with the initial dose for each arm. These data may help inform development of future long-acting SARS-CoV-2 mAbs.

**Figure:**
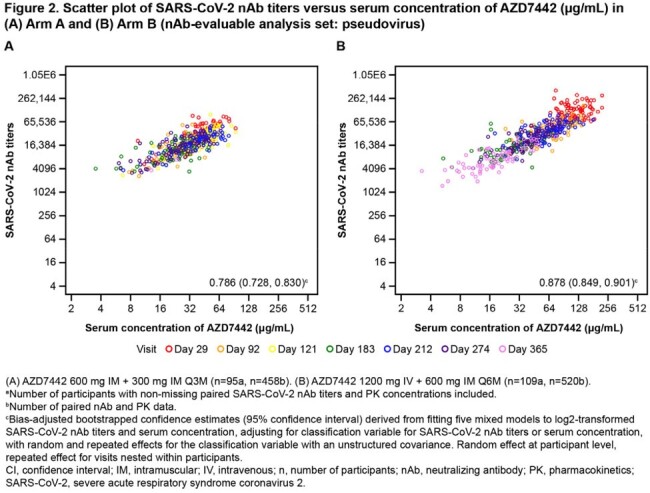

**Disclosures:**

Taylor Cohen, PhD, AstraZeneca: Employee, holds or may hold stock Chigo Munthali, MBBS, MSc, DPM, MFPM, CCT(Pharma Med.), AstraZeneca: Employee, holds or may hold stock Herve Tchouakam Kouekam, MSc, Cytel Inc. (contracted to AstraZeneca): Employee of Cytel Inc. Sam Matthews, MSc, Exploristics Ltd. (contracted to AstraZeneca): Employee of Exploristics Ltd. Audrey Sharbaugh, PhD, AstraZeneca: Employee, holds or may hold stock Rohini Beavon, PhD, AstraZeneca: Employee, holds or may hold stock Dilki Wickramarachchi, PhD, AstraZeneca: Employee, holds or may hold stock Sharon Otal, HBSc, MBA, AstraZeneca: Employee, holds or may hold stock Haitao Yang, PhD, AstraZeneca: Employee, holds or may hold stock Anastasia A. Aksyuk, PhD, AstraZeneca: Employee, holds or may hold stock Lindsay E. Clegg, PhD, AstraZeneca: Employee, holds or may hold stock Huixia Zhang, PhD, AstraZeneca: Employee, holds or may hold stock Antonella Nadia Tuillio, MD, AstraZeneca: Employee, holds or may hold stock Seth Seegobin, PhD, AstraZeneca: Employee, holds or may hold stock Katie Streicher, PhD, AstraZeneca: Employee of AstraZeneca and may own AstraZeneca stock or stock options. David Wheeler, MD, AstraZeneca: Funding for clinical research|Gilead: Funding for clinical research|Janssen: Funding for clinical research Lee-Jah Chang, MD, AstraZeneca: Employee of AstraZeneca Hugh Montgomery, MD, AstraZeneca: Advisor/Consultant|Millfield Medical Ltd.: Advisor/Consultant|Millfield Medical Ltd.: Consulted for Millfield Medical Ltd during the development of a new Closed Circuit Continuous Positive Airway Pressure machine.|UK NIHR BRC at University College London Hospitals: Funding

